# IRE1α Promotes Zika Virus Infection via XBP1

**DOI:** 10.3390/v12030278

**Published:** 2020-03-03

**Authors:** Elena P. Kolpikova, Ana R. Tronco, Andreas B. Den Hartigh, Konner J. Jackson, Takao Iwawaki, Susan L. Fink

**Affiliations:** 1 Department of Laboratory Medicine, University of Washington, Seattle, WA 98195, USA; 2 Division of Cell Medicine, Department of Life Science, Medical Research Institute, Kanazawa Medical University, Uchinada, Ishikawa 920-0265, Japan

**Keywords:** Zika virus, flavivirus, endoplasmic reticulum, ER stress, unfolded protein response, IRE1α, XBP1

## Abstract

Zika virus (ZIKV) is an emergent member of the *Flaviviridae* family which causes severe congenital defects and other major sequelae, but the cellular processes that support ZIKV replication are incompletely understood. Related flaviviruses use the endoplasmic reticulum (ER) as a membranous platform for viral replication and induce ER stress during infection. Our data suggest that ZIKV activates IRE1α, a component of the cellular response to ER stress. IRE1α is an ER-resident transmembrane protein that possesses a cytosolic RNase domain. Upon activation, IRE1α initiates nonconventional cytoplasmic splicing of *XBP1* mRNA. Spliced *XBP1* encodes a transcription factor, which upregulates ER-related targets. We find that ZIKV infection induces *XBP1* mRNA splicing and induction of XBP1 target genes. Small molecule inhibitors of IRE1α, including those specific for the nuclease function, prevent ZIKV-induced cytotoxicity, as does genetic disruption of IRE1α. Optimal ZIKV RNA replication requires both IRE1α and XBP1. Spliced XBP1 has been described to cause ER expansion and remodeling and we find that ER redistribution during ZIKV infection requires IRE1α nuclease activity. Finally, we demonstrate that inducible genetic disruption of IRE1α and XBP1 impairs ZIKV replication in a mouse model of infection. Together, our data indicate that the ER stress response component IRE1α promotes ZIKV infection via XBP1 and may represent a potential therapeutic target.

## 1. Introduction

Zika virus (ZIKV) is a mosquito-borne flavivirus that has recently been associated with severe consequences of infection including congenital microcephaly [1,2]. There remains no specific antiviral in clinical use for ZIKV, reflecting a need for a better understanding of the basic biology of this virus [3,4]. Flaviviruses encode only 10 proteins and rely on the manipulation of host cell processes to facilitate their replication cycle. After receptor-mediated entry, endosome acidification releases the positive sense RNA genome, which is translated and replicated. Flaviviruses induce membrane structures derived from the endoplasmic reticulum (ER), which serve as a scaffold for viral replication [5,6]. Viral packaging occurs on the ER and immature virions bud into the ER lumen for transport to the trans-Golgi and subsequent exocytosis. 

Many viruses, including members of the *Flaviviridae* family, perturb the environment within the ER, inducing a state termed ER stress [7,8]. The unfolded protein response (UPR) is a cellular signaling pathway to detect and alleviate ER stress [9,10]. The UPR is initiated by three ER transmembrane proteins: protein kinase receptor-like ER kinase (PERK), activating transcription factor 6 (ATF6), and inositol-requiring enzyme 1 α (IRE1α). ER stress causes IRE1α to undergo oligomerization and autophosphorylation, which activates its cytosolic RNase domain to initiate nonconventional splicing of *XBP1* mRNA. Spliced *XBP1* is a specific product of activated IRE1α and encodes a transcription factor that upregulates targets that are involved in ER function [11]. IRE1α also targets other specific RNAs, leading to their degradation in a process termed regulated IRE1-dependent decay (RIDD) [12,13]. 

The role of IRE1α in infection appears to vary for different members of the *Flaviviridae* family. Hepatitis C virus (HCV) activates IRE1α [14] to promote viral replication [15] independently of XBP1 by preventing apoptotic death of infected cells [16]. Dengue (DENV) and Japanese encephalitis viruses (JEV) also benefit from IRE1α via an XBP1-independent mechanism [17,18,19,20], whereas West Nile virus (WNV) replication is unaffected by either IRE1α [21,22] or XBP1 [23]. Conflicting results have been obtained for tick-borne encephalitis virus, with IRE1α nuclease inhibition either limiting viral replication [24] or having no effect [22]. ZIKV activates IRE1α, as demonstrated by the presence of spliced *XBP1* in ZIKV-infected cultured cells and brain tissue from ZIKV-infected embryonic mice [25,26,27]. In this study, we examined the role of IRE1α in ZIKV infection and found that IRE1α promotes ZIKV replication via XBP1 in cultured cells. We further found that genetic disruption of IRE1α and XBP1 limits ZIKV infection in multiple tissues in vivo in an adult murine infection model. Together, these findings reveal that IRE1α and XBP1 are cellular host factors that promote ZIKV replication, providing insight that could lead to targeted therapeutic intervention.

## 2. Materials and Methods 

### 2.1. Reagents

Cells were treated with 10 μg/mL tunicamycin (Sigma-Aldrich), 100 nM KIRA6 (MilliporeSigma), 50 μM STF-083010 (MilliporeSigma), 25–50 μM 4μ8C (8-formyl-7-hydroxy-4-methylcoumarin, MilliporeSigma), or 25–50 μM AMC (7-amino- 4-methylcoumarin) (VWR). Viability was assessed by quantifying ATP in metabolically active cells using the CellTiter-Glo 2.0 Assay (Promega).

### 2.2. Cells and Viruses

H1-HeLa cells and Vero cells were propagated in high-glucose Dulbecco’s modified Eagle’s medium (DMEM) (Gibco) supplemented with 10% fetal bovine serum (Serum Plus II, MilliporeSigma), 10 mM Hepes, and 50 U/mL penicillin-streptomycin (Gibco). *Aedes albopictus* mosquito (C6/36) cells for viral propagation were maintained at 30 °C in DMEM supplemented with 10% fetal bovine serum, penicillin-streptomycin, and 1% tryptose phosphate broth (Sigma). ZIKV isolate FSS13025 (Cambodia, 2010, Asian lineage) was a gift from R. Tesh (University of Texas Medical Branch at Galveston) and was propagated in C6/36 *Aedes albopictus* cells at 30 °C and titered in Vero cells. HeLa cells were treated with inhibitors for four hours prior to infection with ZIKV at an MOI of 0.01. Mouse adapted ZIKV Dakar strain [28] was a gift from M. Diamond (Washington University in St. Louis) and was propagated for one passage in Vero cells and titered in Vero cells.

IRE1α CRISPR/Cas9 knockdown cells were made by co-transfection of human IRE1α CRISPR/Cas9 KO plasmids and human IRE1α homology directed repair plasmids (Santa Cruz Biotechnology). These plasmids included three separate guide RNAs and their corresponding homology-directed DNA repair templates. Individual clones that incorporated the homology directed repair template were selected with puromycin, harvested with cloning cylinders, and expanded.

XBP1 CRISPR/Cas9 knockdown cells were made by lentiviral transduction of a vector encoding Cas9, puromycin selection marker, and human *XBP1* CRISPR guide RNA 1 or 2 in the pLentiCRISPR v2 backbone (GenScript). Control cells were made by lentiviral transduction of a vector encoding Cas9, puromycin selection marker, and nontargeting control gRNA (sgCtr- LentiCRISPRv2, Addgene plasmid #107402, a gift from William Kaelin) [29]. Transduced clones were isolated by serial dilution, selected with puromycin, and expanded.

### 2.3. Expression Analysis

Total RNA isolated using the Direct-zol RNA Kit (Zymo Research) was used to synthesize complementary DNA (cDNA) using the iScript cDNA Synthesis Kit (Bio-Rad). Quantitative RT-PCR was performed on a Bio-Rad CFX Connect using SYBR Green (Bio-Rad) with the following primers (all primers listed in the 5′ to 3′ orientation): 

*human spliced Xbp1*: TGC TGA GTC CGC AGC AGG TG (forward) and GCT GGC AGG CTC TGG GGA AG (reverse); *human ERDJ4:* TAG TCG GAG GGT GCA GGA TA (forward) and CGC TCT GAT GCC GAT TTT GG (reverse); *human p58IPK:* TGT GTT TGG GAT GCA GAA CTA C (forward) and TCT TCA ACT TTG ACG CAG CTT (reverse); *ZIKV NS5:* GGC CAC GAG TCT GTA CCA AA (forward) and AGC TTC CAC TGC AGT CTT CC (reverse); *ZIKV 1086:* CCG CTG CCC AAC ACA AG; *ZIKV 1162c:* CCA CTA ACG TTC TTT TGC AGA CAT; *human HPRT*: GAC ACT GGC AAA ACA ATG (forward) and ACA AAG TCT GGC TTA TAT CC (reverse); *human GAPDH:* CAA TGA CCC CTT CAT TGA CC (forward) and GAC AAG CTT CCC GTT CTC AG (reverse); *mouse Hprt:* GTT GGA TAC AGG CCA GAC TTT GTT G (forward) and GAG GGT AGG CTG GCC TAT TGG CT (reverse); *mouse Actb:* CAC TGT CGA GTC GCG TCC (forward) and TCA TCC ATG GCG AAC TGG TG (reverse); *ZIKV 1183F:* CCA CCA ATG TTC TCT TGC AGA CAT ATT G; *ZIKV 1268R*: TTC GGA CAG CCG TTG TCC AAC ACA AG [28]; *mouse Xbp1 deleted exon:* CCT GAG CCC GGA GGA GAA (forward) and CTC GAG CAG TCT GCG CTG (reverse); *mouse Ire1 (Ern1) deleted exon:* TGG ACT GGC GGG AGA ACA TC (forward) and GGT CTC TCA CAG AGC CAC CTT (reverse).

Melt curve analysis was used to assess whether single reaction products were produced. For human cell samples, expression was calculated relative to *HPRT*, with equivalent results also obtained relative to *GAPDH*. ZIKV RNA from cell culture was quantified with the *ZIKV NS5* primers [30] and results were confirmed with the *ZIKV 1086, ZIKV 1162c* primer pair [31]. ZIKV RNA from infected mouse tissues was quantified with the *ZIKV 1183F* and *1268R* primers and expression was calculated relative to *Hprt*, with equivalent results also obtained relative to *Actb*. 

### 2.4. Immunofluorescence Microscopy

After fixation in 2% paraformaldehyde, cells were permeabilized with 0.2% Triton-X100 in PBS and blocked with 3% BSA + 0.2% Tween-20 in PBS. Cells were labeled with anti-PDIA3 mouse monoclonal antibody (MilliporeSigma, catalog number AMAB90988), anti-NS4B rabbit polyclonal (Genetex, catalog number GTX133311), anti-phospho IRE1α pSer724 (ThermoFisher, catalog number PA116927), and highly cross-adsorbed donkey anti-mouse Alexa Fluor 488 secondary antibody (Invitrogen, catalog number A-21202) or donkey anti-rabbit Alexa Fluor 555 secondary antibody (Invitrogen, catalog number A-31572 all diluted in 3% BSA + 0.2% Tween-20 in PBS. TO-PRO-3 (Invitrogen. Catalog number T3605) was used to label nuclei. 

For phospho-IRE1α staining, three random high-powered fields were collected for each condition using identical capture settings for the target of interest on the Leica SP8X confocal microscope at the UW W.M. Keck Microscopy Center. Phospho-IRE1α staining intensity was measured for each cell using Image J version 1.51f and the percentage of cells above the baseline threshold was calculated for each image with a minimum total cell count of 200 cells for each condition. For PDI staining, five random high-powered fields were collected for each condition using identical capture settings for the target of interest. Cells with condensed ER morphology (ER reorganization) were quantified by visual inspection for each image with a minimum total cell count of 300 cells for each condition. 

### 2.5. Western Blot

Cells were collected with trypsin and pelleted together with non-adherent cells. Cell pellets were washed with PBS and digested with Protein Extraction Reagent Type 4 (MilliporeSigma) with added HALT protease inhibitor cocktail (ThermoFisher), PMSF protease inhibitor (ThermoFisher), and Benzonase nuclease (MilliporeSigma) on ice for 25 min, mixed with loading buffer, and heated at 95 °C for 10 min under reducing conditions. Proteins were separated by SDS-polyacrylamide gel electrophoresis using Any kD TGX stain free gels (BioRad) and transferred to nitrocellulose membranes. Membranes were probed with anti-vinculin mouse monoclonal antibody (Santa Cruz Biotechnology, catalog number sc-73614) and either rabbit polyclonal anti-Zika virus NS4B (GeneTex, catalog number GTX133311) or rabbit polyclonal XBP1 (Invitrogen, catalog number PA5-27650), followed by incubation with secondary antibodies, donkey anti-rabbit IRDye 800CW (LI-COR, catalog number 926-32213), and goat anti-mouse IRDye 680RD (LI-COR, catalog number 926-68070). The blots were imaged with an Odessey Infrared Imaging System (LI-COR Biosciences) and relative density units were calculated with Image Studio Lite version 5.2 and normalized to vinculin.

### 2.6. Plaque Assay

Vero cells were cultured in 6-well plates to confluency and infected with serially-diluted samples for 1 h with shaking every 15 min. Inhibitors in the supernatant samples were diluted to ineffective concentrations. The inoculum was removed and replaced with overlay containing supplemented high-glucose DMEM and 0.5% each SeaPlaque and SeaKem agarose (Lonza). After 5 days, monolayers were fixed and stained with crystal violet and plaques were counted visually. 

### 2.7. Mouse Model of ZIKV Infection

4 to 6 week-old *Xbp1*^flox/flox^ *Ern1*^flox/flox^ ESR Cre+ and Cre− littermate mice were given 5 consecutive daily doses of 75 mg/kg tamoxifen (MilliporeSigma) in corn oil intraperitoneally (i.p.) to induce expression of Cre recombinase. After resting for 3 days, mice were infected i.p. with 1E4 PFU of mouse adapted ZIKV Dakar [28]. Animals received 1.5 mg interferon receptor blocking monoclonal antibody MAR1-5A3 (Leinco, catalog number I-401) i.p. the day prior to infection and 1 mg the day after infection. Mice were euthanized 3 days after infection. Harvested tissues were immediately frozen on dry ice and stored at −80 °C until processing. Samples were homogenized in TRI Reagent (Zymo Research) with Lysing Matrix D beads (MP Biomedicals) on a FastPrep tissue homogenizer (MP Biomedicals). All procedures performed in this study were approved by the University of Washington Institutional Animal Care and Use Committee (28 December 2016). 

### 2.8. Statistics

The unpaired two-tailed Student’s *t* test or the Mann-Whitney test were used for comparisons between two groups. *P* values of less than 0.05 were considered statistically significant.

## 3. Results

### 3.1. Zika Virus Infection Stimulates IRE1α Activation and Induction of XBP1 Targets

**Figure 1:**
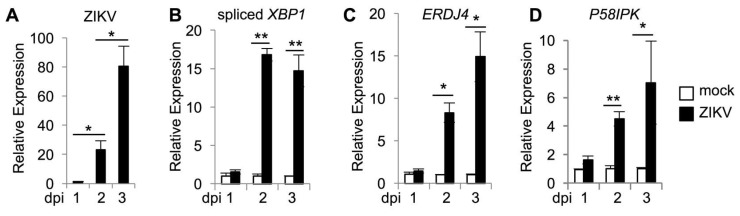
ZIKV infection activates IRE1α and induction of XBP1 targets. Cells were infected with ZIKV and RNA was harvested at the indicated number of days post infection (dpi). The relative mRNA abundance of ZIKV RNA (**A**) spliced *XBP1* (**B**), *ERDJ4* (**C**), and *P58IPK* (**D**) were determined by quantitative RT-PCR. Data are means ± SD of four replicates and are representative of at least two independent experiments. * *p* < 0.05, ** *p* < 0.001, by unpaired *t* test.

Epithelial cells represent a target for ZIKV after both vector borne and sexual transmission [32]. We infected human HeLa epithelial cells with an Asian lineage, patient-derived strain of ZIKV (FSS13025) at an MOI of 0.01 and observed an increase in viral RNA over the course of infection (Figure 1A). We confirmed prior observations of ZIKV-induced IRE1α activation [25,26,27] in this system, with qRT-PCR using primers specific for spliced *XBP1* mRNA [33] (Figure 1B). In addition, we observed an increase in phosphorylated IRE1α in ZIKV infected cells (Figure S1A,B), and increased production of the protein product of spliced *XBP1* (Figure S1C,D). 

HCV and WNV activate IRE1α to initiate *XBP1* mRNA splicing, but downstream effects of XBP1 are blocked and XBP1 targets are not transcribed [14,34,35]. Gene expression changes that are broadly characteristic of an ER stress response have been observed during ZIKV infection [25,26], but there is overlap among transcriptional changes induced by the three UPR sensors [36]. To identify XBP1 specific targets, we examined gene induction in response to the ER stress inducing agent, tunicamycin. Published data demonstrated that expression of *ERDJ4* and *P58IPK* is XBP1-dependent [37] and consistently, we found that induction of *ERDJ4* (Figure S2A) and *P58IPK* (Figure S2B) requires XBP1 in tunicamycin treated cells. Although XBP1 targets are not induced in response to HCV and WNV [34,35], we found that ZIKV infection does induce the XBP1 targets *ERDJ4* (Figure 1C) and *P58IPK* (Figure 1D). Together, these results suggest that ZIKV infection activates IRE1α to splice *XBP1* mRNA, leading to upregulation of XBP1 targets.

### 3.2. IRE1α Inhibitors Prevent Zika-Induced Cell Death

**Figure 2:**
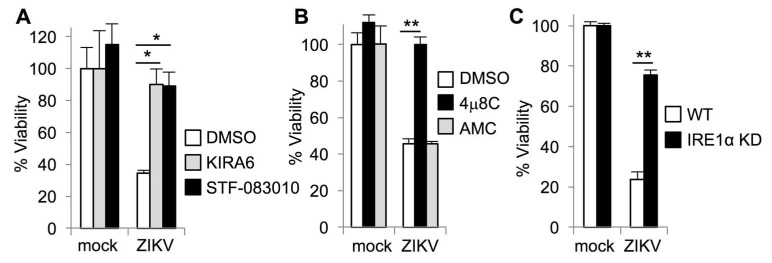
IRE1α inhibitors prevent ZIKV-induced cell death. (**A**+**B**) Cells were treated with small molecule inhibitors or DMSO solvent control prior to infection with ZIKV. Viability was measured four days post-infection by quantifying ATP in metabolically active cells. (**A**) The IRE1α kinase inhibitor KIRA6 and nuclease inhibitor STF-083010 prevent loss of viability during ZIKV infection. (**B**) The IRE1α nuclease inhibitor 4μ8C, but not AMC, a structurally similar negative control compound, prevents ZIKV-induced loss of viability. (**C**) Wildtype (WT) and IRE1α knockodown (KD) cells were infected with ZIKV and viability was measured three days post-infection. Data are means ± SD of three replicates and are representative of at least two independent experiments. * *p* < 0.01, ** *p* < 0.001, by unpaired *t* test.

We recently found that IRE1α promotes HCV replication by preventing apoptotic death of infected cells [16]. Based on these findings, we hypothesized that IRE1α could similarly promote viability during ZIKV infection and blocking IRE1α would sensitize ZIKV-infected cells to die. To test this hypothesis, we measured the viability of ZIKV-infected cells in the presence of the IRE1α kinase inhibitor, KIRA6 [38]. In contrast to our observations with HCV, we found that ZIKV-infected cells underwent cell death and KIRA6 prevented ZIKV-induced cytotoxicity (Figure 2A). The kinase activity of IRE1α mediates autophosphorylation to activate the RNase domain, but can also activate other signaling pathways such as c-Jun amino-terminal kinase (JNK) [39]. To determine whether IRE1α RNase activity contributed to ZIKV-induced cell death, we treated cells with the selective IRE1α nuclease inhibitor, STF-083010 [40]. We found that STF-083010 was also protective against ZIKV-induced cytotoxicity (Figure 2A). To further verify this observation, we used a distinct IRE1α nuclease inhibitor 4μ8C (8-formyl-7-hydroxy-4-methylcoumarin) [41] and found that 4μ8C also prevented the death of ZIKV-infected cells (Figure 2B). As a negative control, the structurally similar compound AMC (7-amino-4-methylcoumarin) [16] had no effect at an equimolar concentration (Figure 2B). To determine whether genetic disruption of IRE1α would recapitulate the results obtained with small molecule inhibitors, we genetically disrupted IRE1α using CRISPR-Cas9. We found that IRE1α genetic disruption reduced the death of ZIKV-infected cells (Figure 2C). Together, these results suggest that IRE1α inhibition and genetic disruption reduce ZIKV-induced cytotoxicity, in contrast to our observations with HCV infection.

### 3.3. IRE1α and XBP1 Promote Zika Virus Replication in Cultured Cells

**Figure 3:**
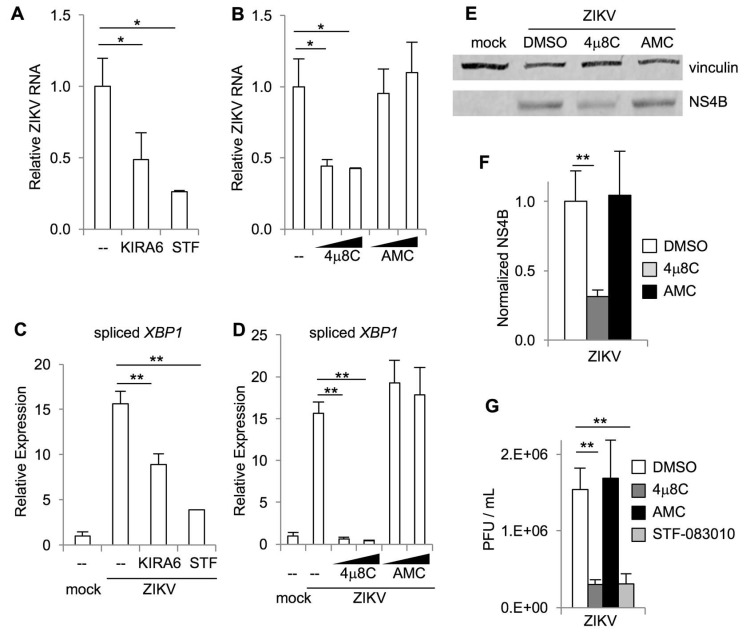
IRE1α inhibitors reduce ZIKV replication. Cells were treated with small molecule inhibitors or DMSO solvent control prior to infection with ZIKV. RNA was harvested two days post-infection and the relative abundance of ZIKV RNA (**A**+**B**) and spliced *XBP1* mRNA (**C**+**D**) were determined by quantitative RT-PCR. Data are means ± SD of three replicates and are representative of at least two independent experiments. The relative abundance of viral NS4B and vinculin loading control in cell lysates two days post-infection was determined by Western blotting and densitometry (**E**). The ratio of sXBP1 to vinculin is shown, normalized to uninfected cells (**F**). Data are means ± SD of three independent experiments. Viral titers in the cell culture medium were measured by plaque assay (**G**). PFU, plaque-forming units. Data are means ± SD of six replicates and are representative of three independent experiments. * *p* < 0.05, ** *p* < 0.01, by unpaired *t* test.

Our results suggest that IRE1α promotes ZIKV-induced cell death, which could be secondary to an effect on viral replication. To test this hypothesis, we measured ZIKV RNA in infected cells using qRT-PCR. We found that the IRE1α kinase inhibitor KIRA6 and nuclease inhibitor STF-083010 both reduced viral RNA in infected cells (Figure 3A). Similarly, the nuclease inhibitor 4μ8C, but not the negative control AMC, reduced viral RNA in infected cells (Figure 3B). All three IRE1α inhibitors prevented *XBP1* splicing (Figure 3C,D). 

In addition to reduction in viral RNA, we found that the IRE1α nuclease inhibitor 4μ8C, but not the negative control AMC, reduced viral NS4B protein in infected cells (Figure 3E,F) and the release of infectious virus detected by plaque assay (Figure 3G). We confirmed this result using the IRE1α nuclease inhibitor STF-083010, which also reduced viral titers in the cell culture medium (Figure 3G).

**Figure 4:**
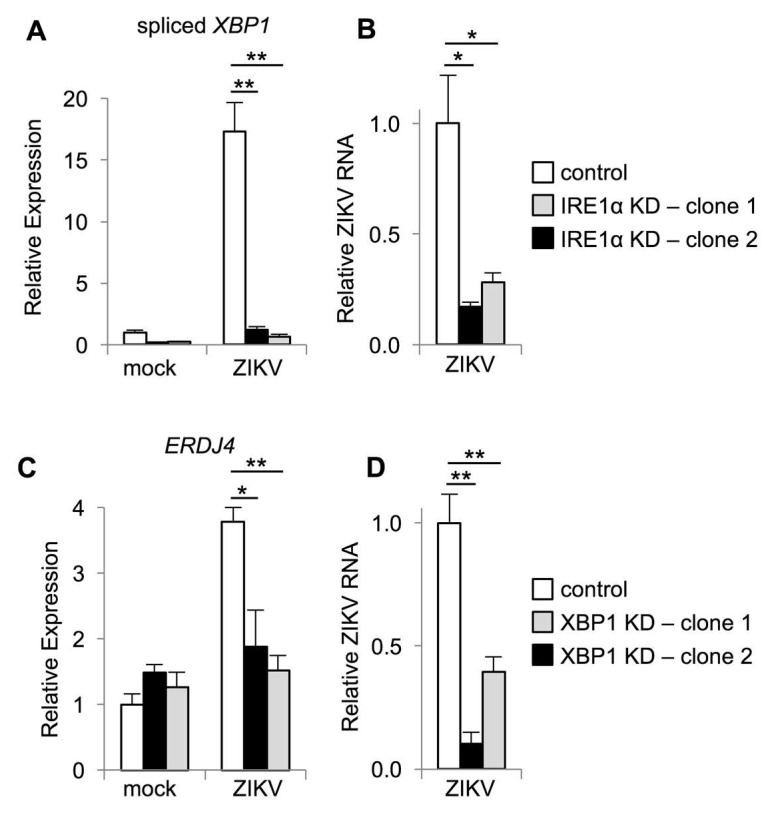
IRE1α and XBP1 promote ZIKV replication. (**A**–**D**) IRE1α or XBP1 knockdown (KD) cells generated using CRISPR-Cas9 were infected with ZIKV and RNA was harvested two days post-infection. The relative abundance of spliced *XBP1* mRNA (**A**), ZIKV RNA (**B**+**D**), and *ERDJ4* mRNA (**C**) were determined by quantitative RT-PCR. Data are means ± SD of three replicates and are representative of at least two independent experiments. *p < 0.05, **p < 0.01, by unpaired *t* test.

To verify these results, we infected cells in which IRE1α was disrupted using CRISPR-Cas9. As a functional control for IRE1α inactivation, we found that that *XBP1* splicing was abolished in two distinct clones of IRE1α knockdown cells, with a reduction of 93–96% (Figure 4A). Consistent with our observations using IRE1α small molecule inhibitors, we found reduced ZIKV RNA in IRE1α knockdown cells (Figure 4B). Together, these results suggest that IRE1α promotes ZIKV replication and subsequent viral cytopathic effect.

STF-083010 and 4μ8C are specific for the nuclease activity of IRE1α, suggesting that this enzymatic function supports ZIKV replication. IRE1α is an RNase with dual functions to mediate splicing of *XBP1* mRNA and degradation of other RNAs. To determine whether IRE1α promotes ZIKV infection via *XBP1* splicing and activation, we targeted XBP1 for genetic inactivation using CRISPR-Cas9. We found a 73%–92% reduction in spliced *XBP1* in these cells. As a functional control for XBP1 inactivation, we found that ZIKV-induced expression of the XBP1 target *ERDJ4* was abolished in these cells (Figure 4C). We found reduced ZIKV RNA in XBP1 knockout cells generated using two separate guide RNAs (Figure 4D), similar to our observations with IRE1α inactivation. Together, these results suggest that IRE1α promotes ZIKV replication via XBP1.

**Figure 5:**
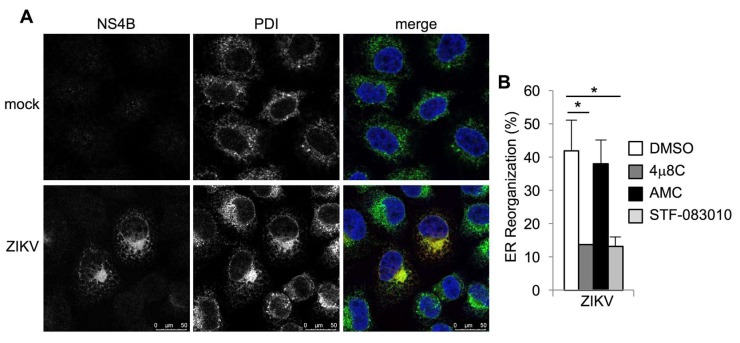
IRE1α inhibitors prevent ZIKV-induced ER reorganization. (**A**+**B**) Cells were infected with ZIKV for two days. Viral NS4B protein (red in merged image) and the ER marker, protein disulfide isomerase (PDI, green in merged image), were visualized by immunostaining. Nuclei were counterstained with TO-PRO-3 (blue). (**B**) Cells were treated with small molecule inhibitors or DMSO solvent control prior to infection with ZIKV and PDI staining, and ER reorganization was quantified. Data are means ± SD of three independent experiments. * *p* < 0.05, by unpaired *t* test.

Spliced *XBP1* is required and sufficient to cause ER expansion and remodeling in specialized secretory cells [42,43,44]. Flaviviruses, including ZIKV, induce ER expansion and redistribution, which creates membranous platforms for viral RNA replication and virion packaging [45,46]. To determine whether ER redistribution in ZIKV-infected cells requires IRE1α, we performed immunostaining for the ER marker, protein disulfide isomerase (PDI). Consistent with other studies [45,46,47], we found striking PDI redistribution in ZIKV-infected cells with perinuclear ER accumulation (Figure 5A). PDI redistribution was abolished by the IRE1α nuclease inhibitor 4μ8C, but not the structurally similar negative control, AMC (Figure 5B and Figure S3). The IRE1α nuclease STF-083010 similarly prevented PDI redistribution in ZIKV-infected cells (Figure 5B and Figure S3). Together, these results suggest that ER redistribution during ZIKV infection requires IRE1α nuclease activity.

### 3.4. IRE1α and XBP1 Promote Zika Virus Infection in Mice

These data demonstrate that IRE1α and XBP1 are required for optimal ZIKV replication in cultured cells and we hypothesized that these host factors would also contribute to ZIKV infection in vivo in a mouse model. Type I interferon (IFN α/β) is critical for the control of many flaviviral infections [48]. Blockade of IFN-α/β signaling with the type I interferon receptor blocking monoclonal antibody MAR1-5A3 permits nonlethal ZIKV infection in mice [49]. This model mimics some aspects of human infection with efficient viral replication in the testes of male mice [28,50,51] and infection of the eye causing ocular disease [52].

**Figure 6:**
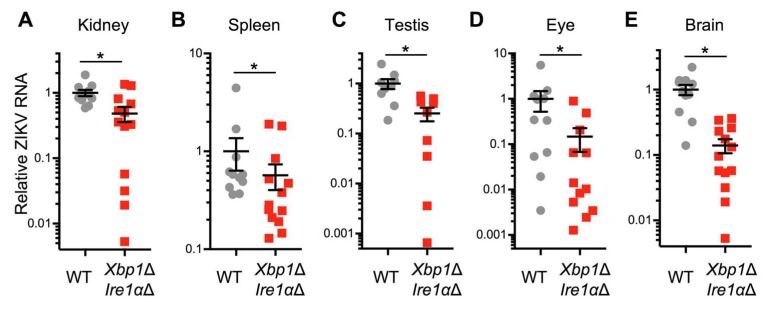
Genetic disruption of IRE1α and XBP1 reduces ZIKV infection in a mouse model. *Xbp1*^flox/flox^ *Ern1*^flox/flox^ ESR Cre+ (*Xbp1*Δ *Ire1α*Δ) or Cre− littermate (WT) mice were treated with tamoxifen to induce the expression of Cre recombinase. Mice were given interferon receptor blocking MAR1-5A3 antibody the day before and after ZIKV infection. RNA was harvested from (**A**) kidney, (**B**) spleen, (**C**) testis, (**D**) eye, and (**E**) brain three days post-infection. ZIKV RNA was measured by quantitative RT-PCR and normalized to *Hprt*. Values represent mean ± SEM involving Cre- (*n* = 11) and Cre+ (*n* = 13) mice pooled from two independent experiments. Testes were obtained from the subset of mice that were male (*n* = 9) for both Cre- and Cre+ animals. *p < 0.05 by Mann-Whitney test.

To determine whether the IRE1α-XBP1 branch of the UPR promotes ZIKV replication in vivo, we used mice in which Cre-recombinase mediated *Xbp1* and *Ern1* (encoding IRE1α) knockout is inducible with tamoxifen [16]. We treated *Xbp1*^flox/flox^ *Ern1*^flox/flox^ ESR Cre positive (*Xbp1*Δ *Ire1*αΔ) and Cre negative littermate control mice with tamoxifen and observed consistent disruption of both genes (Figure S4). We treated these animals with interferon receptor blocking MAR1-5A3 antibody and infected them with a mouse-adapted strain of ZIKV [28]. We measured viral RNA in harvested organs three days post-infection using qRT-PCR. We found reduced ZIKV RNA in the kidney (Figure 6A), spleen (Figure 6B), testis (Figure 6C), eye (Figure 6D), and brain (Figure 6E) of Cre positive (*Xbp1*Δ *Ire1*αΔ) animals compared to Cre negative littermate controls. Together, these data support the hypothesis that IRE1α and XBP1 contribute to ZIKV replication not only in cultured cells, but also in an adult animal model of infection.

## 4. Discussion

Here, we examined the role of the IRE1α-XBP1 branch of the UPR in ZIKV infection (Figure S5). Consistent with published studies demonstrating the presence of spliced *XBP1* mRNA in infected human neural stem cells and brain of ZIKV-infected mice [25,26], we found that ZIKV activates IRE1α to splice *XBP1* mRNA in cultured epithelial cells. We also found that specific XBP1 target genes are upregulated during ZIKV infection. Although we do not yet know how ZIKV activates IRE1α and XBP1, specific proteins from other members of the *Flaviviridae* family regulate this branch of the UPR. For example, HCV non-structural protein NS4B and WNV NS4A and NS4B proteins are each sufficient to trigger IRE1α activation and *XBP1* splicing [34,35,53]. Nonstructural protein NS2B-3 from DENV, but not JEV, has also been reported to stimulate *XBP1* splicing [18].

In contrast to our previous findings with HCV infection [16], IRE1α activity did not prevent the death of ZIKV infected cells, but instead promoted ZIKV-induced cytotoxicity. We hypothesize that reduction in the cytopathic effect with IRE1α inhibition or genetic disruption may be secondary to impaired viral replication. Our results indicate that IRE1α and XBP1 are required for optimal ZIKV replication, both in cultured cells and in a mouse model of infection. This conclusion is based on the measurement of viral RNA, protein, and titer using both inhibitors as well as genetic disruption, with two independent genetically disrupted clones for each gene. We did not consistently observe a difference between the two IRE1α and XBP1 knockout clones and the small differences in viral RNA between the clones (Figure 4) represents experimental variability. The requirement for IRE1α and XBP1 sets ZIKV apart from other flaviviruses as DENV and JEV benefit from IRE1α via an XBP1-indepdendent mechanism [17,18,19,20] and WNV is unaffected by the disruption of either IRE1α or XBP1 [21,22,23].

Based on our findings, we hypothesize that XBP1 target genes promote ZIKV replication. XBP1 is a transcription factor of which its targets include components of ER-associated degradation (ERAD), a pathway that retrotranslocates proteins through the ER membrane to the cytosol for ubiquitination and proteasomal degradation [54]. ERAD components have been identified in screens for host factors that promote flaviviral infection [55,56,57]. However, knockout of ERAD genes had minimal effect on ZIKV infection [55,56], suggesting that other XBP1 targets may promote ZIKV infection. In addition to ERAD components, XBP1 also upregulates targets that facilitate ER expansion and redistribution to accommodate protein production in secretory exocrine cells and plasma cells [42,43]. We found that IRE1α nuclease activity promotes ER redistribution during ZIKV infection. Future studies will be needed to determine whether XBP1-dependent genes directly promote the formation of the ER-based platform for ZIKV replication or if IRE1α and XBP1 contribute to viral replication via another mechanism and the effect on ER redistribution is a secondary consequence of reduced viral RNA and protein.

In this study, we have focused on the IRE1α-XBP1 branch of the UPR, but ZIKV also activates the PERK [26] and ATF6 [25] branches as well. ATF4 downstream of PERK contributes to defects in neurogenesis in the setting of mutation of a critical neuronal protein [58]. Administration of a PERK inhibitor was reported to correct cortical neurogenesis during ZIKV infection without affecting viral replication, suggesting that PERK and ATF4 activation may contribute to fetal microcephaly associated with congenital ZIKV infection [26]. This mechanism may synergize with other described pathways, mediating ZIKV-associated pathogenesis, such as abnormal placental development from exposure to type I interferon [59] and apoptosis of neural progenitor cells [60].

There is emerging evidence for a critical role of IRE1α in diseases including cancer and diabetes and IRE1α inhibitors are under evaluation as potential therapeutics [61]. In preclinical models, these drugs have been well-tolerated and provided robust in vivo inhibition of IRE1α [38,62,63,64]. Given the role of IRE1α in promoting replication of not only ZIKV, but also HCV, DENV, and JEV, we propose the potential novel application of these drugs to treat multiple viral infections. In addition, further understanding the mechanism by which IRE1α and XBP1 contribute to ZIKV replication will provide insight into the basic biology of this important human pathogen.

## Supplementary Materials

The following are available online at https://www.mdpi.com/1999-4915/12/3/278/s1, Figure S1. ZIKV infection induces IRE1α phosphorylation and sXBP1 protein production, Figure S2. ER stress induced expression of ERDJ4 and P58IPK requires XBP1, Figure S3. IRE1α inhibitors limit ZIKV-induced ER reorganization, Figure S4. Efficiency of conditional genetic deletion, Figure S5. Model for the Role of IRE1α and XBP1 during ZIKV Infection.
