# Supplementary material for: IRE1α Promotes Zika Virus Infection via XBP1
Source: Viruses. 2020 Mar 3;12(3):278. doi: 10.3390/v12030278 (PMC7150863; doi:10.3390/v12030278)
Supplement: Supplementary file 1 [file viruses-12-00278-s001.pdf]

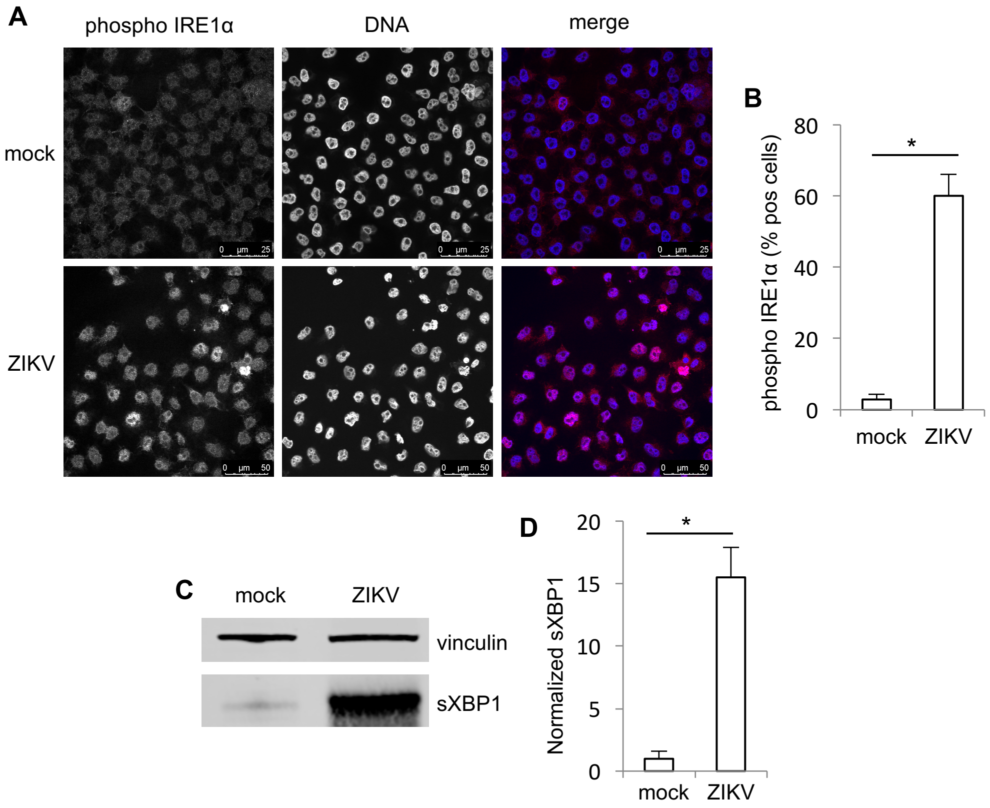

Figure S1. ZIKV infection induces IRE1α phosphorylation and sXBP1 protein production. Cells were infected with ZIKV for two days and phosphorylated IRE1α (Ser724) was visualized by immunostaining (red in merged image) (**A**). Nuclear DNA was counterstained with TO-PRO-3 (blue in merged image). Per cell fluorescence intensity was measured and the percentage of positive cells was quantified (**B**). Data are means ± SD of three fields with a minimum of 60 cells per field, and are representative of at least two independent experiments. The relative abundance of sXBP1 and vinculin loading control in cell lysates three days post-infection was determined by Western blotting and densitometry (**C**). The ratio of sXBP1 to vinculin is shown, normalized to uninfected cells (**D**). Data are means ± SD of three independent experiments. *$P < 0.01$, by unpaired t test.

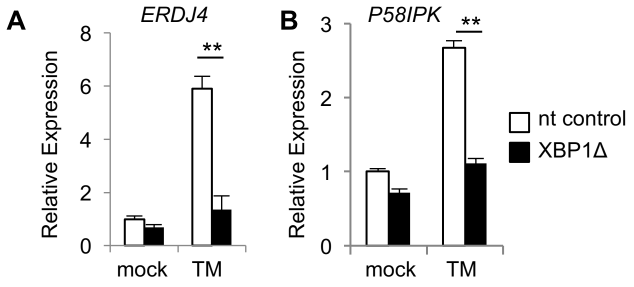

Figure S2. ER stress induced expression of ERDJ4 and P58IPK requires XBP1. Cells transduced with LentiCRISPR vectors encoding XBP1-targeting gRNA (XBP1Δ) or non-targeting (nt) control were treated with tunicamycin (TM) for 5 h. The relative mRNA abundance of *ERDJ4* (**A**) and *P58IPK* (**B**) were determined by quantitative RT-PCR. Data are means ± SD of three replicates and are representative of at least two independent experiments. **P < 0.001, by unpaired t test.

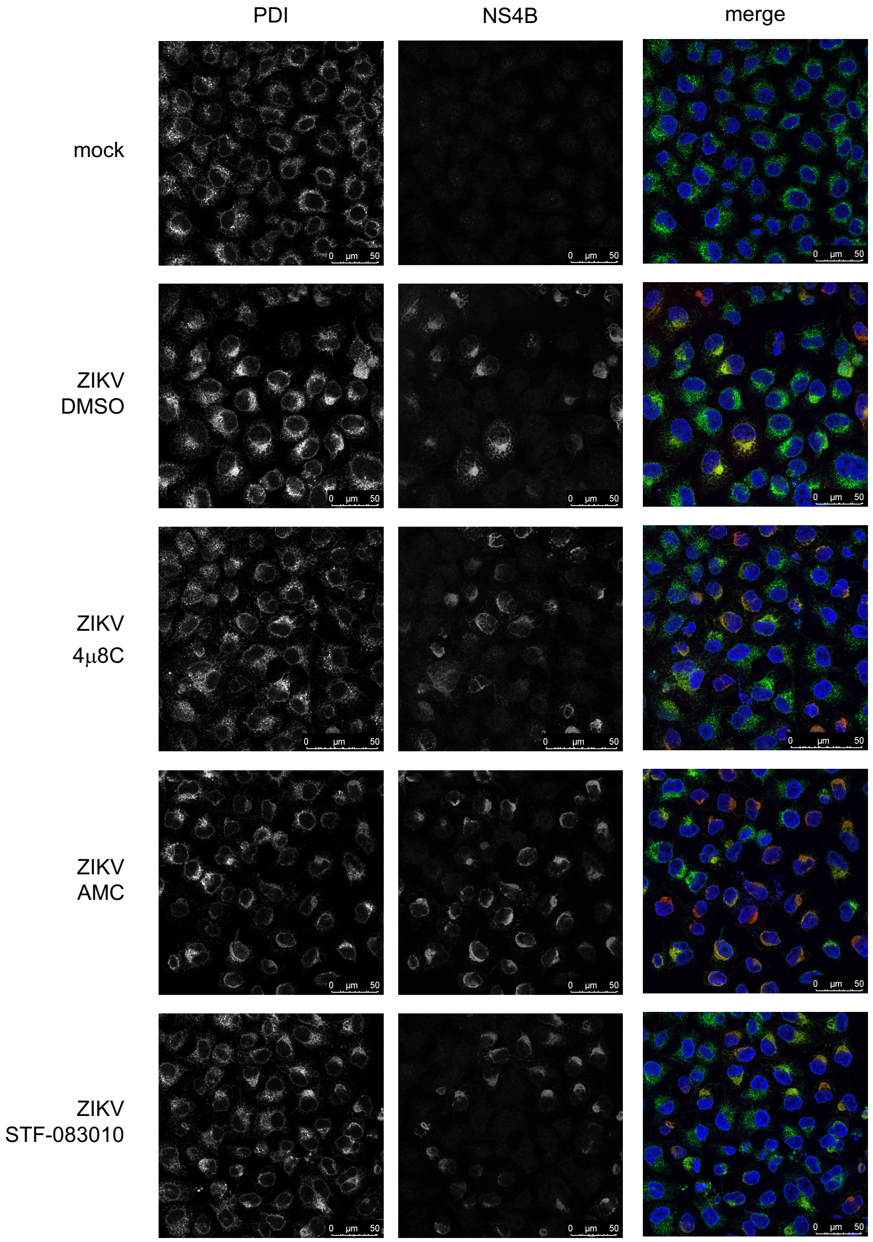

Figure S3. IRE1α inhibitors limit ZIKV-induced ER reorganization. Cells were treated with small molecule inhibitors or DMSO solvent control prior to infection with ZIKV. The ER marker, protein disulfide isomerase (PDI, green in merged image) and viral NS4B protein (red in merged image) were visualized by immunostaining. Nuclei were counterstained with TO-PRO-3 (blue). Representative images from one of three independent experiments are shown.

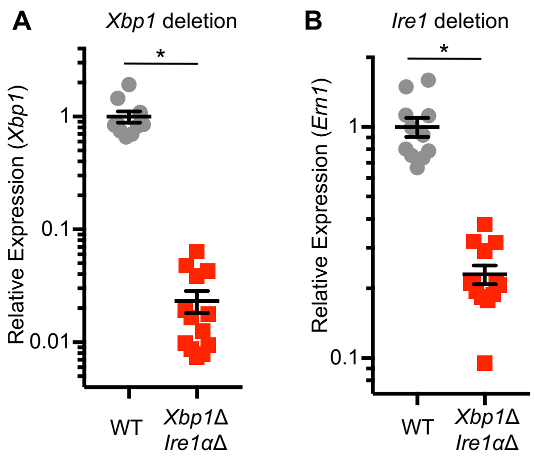

Figure S4. Efficiency of conditional genetic deletion. *Xbp1*$^{flox/flox}$ *Ern1*$^{flox/flox}$ ESR Cre+ (*Xbp1*Δ *Ire1α*Δ) or Cre− littermate (WT) mice were treated with tamoxifen to induce expression of Cre recombinase. Mice were given interferon receptor blocking MAR1-5A3 antibody the day before and after ZIKV infection. RNA was harvested from kidneys 3 days post-infection. Efficiency of Cre recombinase–mediated deletion of *Xbp1* and *Ern1* with was assessed by quantitative RT-PCR using primers binding within the loxP-flanked exons. *$P < 0.05$ by Mann-Whitney test.

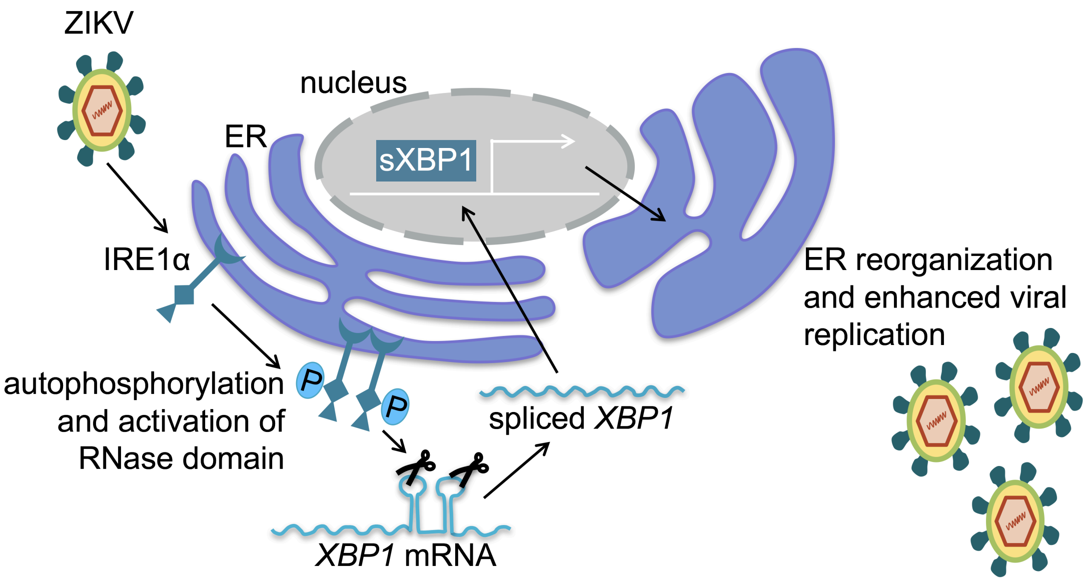

Figure S5. Model for the Role of IRE1α and XBP1 During ZIKV Infection. ZIKV infection activates IRE1α, which triggers its oligomerization, auto-phosphorylation and activation of its RNase domain. Active IRE1α processes *XBP1* mRNA and spliced *XBP1* encodes a transcription factor. XBP1 activity contributes to ER reorganization and enhancement of viral replication.
